# Resting-state EEG gamma power predicts immediate and delayed recall in healthy adults

**DOI:** 10.1007/s11571-025-10313-2

**Published:** 2025-08-26

**Authors:** Valerio Manippa, Giorgia Francesca Scaramuzzi, Gaetano Scianatico, Ester Cornacchia, Anna Concetta Spina, Paolo Taurisano, Giancarlo Logroscino, Davide Rivolta

**Affiliations:** 1 Department of Education, Psychology and Communication, University of Bari Aldo Moro, Bari, Italy; 2 Center for Neurodegenerative Diseases and the Aging Brain, University of Bari Aldo Moro at Pia Fondazione “Cardinale G. Panico”, Lecce, Italy; 3 Department of Translational Biomedicine and Neurosciences (DiBraiN), University of Bari Aldo Moro, Bari, Italy; 4 Department of Precision and Regenerative Medicine and Ionian Area, University of Bari Aldo Moro, Bari, Italy

**Keywords:** Psychophysiology, Brain waves, Cognition, Neurophysiology, Learning

## Abstract

**Objective:**

Resting-state EEG (rsEEG) provides insights into neural mechanisms underlying memory by reflecting intrinsic brain activity. This study tested whether rsEEG spectral power and theta-gamma phase-amplitude coupling (PAC) can predict memory performance in healthy adults.

**Methods:**

Twenty-four healthy adults participated in two rsEEG recording sessions, followed by memory tests assessing multimodal Working Memory (WM), Immediate Recall (IR), and Delayed Recall (DR). The predictive value of rsEEG spectral power across frequency bands and theta-gamma PAC was analyzed in relation to memory performance.

**Results:**

High-gamma (h-γ, 51–100 Hz) power significantly predicted IR and DR, accounting for over 43% of the variance. Temporal and frontal h-γ power positively correlated with memory performance, while posterior h-γ power showed a negative correlation. Temporal low-gamma (30–49 Hz) power positively predicted DR, and posterior and frontal theta power was significantly linked to IR. Other frequency bands showed marginal associations, and theta-gamma PAC had limited predictive value.

**Conclusions:**

Spontaneous gamma activity emerged as a key predictor of memory performance in healthy adults, highlighting the role of brain networks in encoding and retrieval processes.

**Supplementary Information:**

The online version contains supplementary material available at 10.1007/s11571-025-10313-2.

## Significance

These findings identify gamma activity as a potential biomarker for memory function and offer a foundation for future research into neuromodulation strategies and early detection of cognitive decline.

**Supplementary Information:**

The online version contains supplementary material available at 10.1007/s11571-025-10313-2.

## Introduction

Memory is a multifaceted cognitive process involving various storage types and processes. Working Memory (WM) allows temporary storage and manipulation of information within short-term memory (STM) and supports tasks like language comprehension and reasoning (Baddeley 1992; Jabès et al. 2021). Immediate recall (IR) refers to retrieving information immediately after its presentation, relying on attentional and encoding processes that support both STM and long-term memory (LTM) (Baddeley 2012). In contrast, delayed recall (DR) involves recalling learned information after a delay, reflecting LTM consolidation and retrieval processes (Squire 2004). Thus, WM and IR depend on temporary storage, whereas DR indicates the effectiveness of memory consolidation and retrieval (Tulving 1985).

Lately, since memory issues are typically the first complaints reported by Mild Cognitive Impairment (MCI) and Alzheimer Disease (AD) patients (2022 Alzheimer’s disease facts and figures, 2022; Petersen 2016), there has been a growing interest in the neurophysiological features of memory impairment (Cuevas et al. 2012). A widely used approach is the recording of neural oscillations during rest (i.e., resting state EEG, rsEEG), providing insights into spontaneous brain activity supporting cognitive functioning (Wan et al. 2023). Moreover, rsEEG allows detection of neural markers of healthy aging and functional changes in the progression to neurodegenerative processes (Liu et al. 2022; Rossini et al. 2007).

Accordingly, rsEEG reflects age-related cognitive impairment (Anderson and Perone 2018): Jabès et al. (2021) observed theta (5–7 Hz) and alpha (8–12 Hz) power reduction in older adults, indicative of alterations in attentional and inhibitory processes and an overall decline in executive function and memory. Alpha activity reduction may also suggest a shift towards less organized and more random brain functional organization with age (Petti et al. 2016). A substantial body of research links rsEEG aberrations to cortical neurodegeneration and cognitive decline in Mild Cognitive Impairment (MCI) and Alzheimer’s Disease (AD) (Chen et al. 2023): AD patients exhibit slowing activity (Dauwels et al. 2010; Jeong 2004), characterized by increased delta (2–4 Hz) and theta relative power oscillation and decreased alpha, beta (13–29 Hz) and gamma (> 30 Hz) relative power as the disease progresses (Güntekin et al. 2023; Hamm et al. 2015; Perez et al. 2022). Posterior alpha power positively correlates with global cognitive status in AD and MCI patients, with lower alpha power associated with poorer cognitive performance (Luckhaus et al. 2008). However, the specific EEG features underlying the latter, and specifically memory functioning, remain largely unclear.

Studies on the association between rsEEG and memory in healthy individuals produced divergent findings, depending on parameters and measures (Jabès et al. 2021). Theta band activity seems to be a crucial component of WM, positively correlating to cognitive load. WM tasks modulate theta activity in various brain regions like the frontal, hippocampal, and parietal cortex (Moran et al. 2010), linked to a frontotemporal network involving the prefrontal and medial temporal cortex (i.e., hippocampal formation and parahippocampal region), essential in LTM processes (Battaglia et al., 2011). Accordingly, Klimesch et al. (1999) previously found that theta activity in hippocampus-cortical feedback loops is associated with forming new memories (i.e., IR), while upper alpha activity in thalamocortical feedback loops correlates with DR of semantic information. A robust correlation between rsEEG phase-specific activity and semantic LTM scores, emphasizing its role in assessing memory’s neural substrates, emerges in Amin et al. (2025) neurophysiological model, based on rsEEG functional connectivity patterns in theta, alpha, and gamma rhythms.

Specifically, hippocampal gamma oscillations appear central in cognitive functioning and neurocognitive decline (Başar 2013; Mably and Colgin 2018). Gamma activity, generated by parvalbumin-expressing inhibitory interneurons (Bartos et al. 2007), supports higher-order cognitive functions like memory (Kucewicz et al. 2017; Mably and Colgin 2018), being essential for selecting and integrating information across distributed brain networks (Fries 2009; Rivolta et al. 2015; Senkowski et al. 2008). Although rsEEG gamma oscillations appear to be reduced in elders (Murty et al. 2020), increased gamma activity during WM tasks has been observed, possibly reflecting a compensatory mechanism for multisensory integration driven by the need for greater resources (Senkowski et al. 2009). These findings have positioned gamma oscillations as key targets for early diagnosis and therapeutic interventions of neurodegenerative diseases (Manippa et al. 2024a; Naro et al. 2016). Brain activity can be dissected in time and frequency domains, revealing information about power, phase, and interactions between different frequency bands. In this context, inter-frequency coupling (i.e., the interaction between different oscillation frequencies) has gained interest for its potential to integrate neural information in a way relevant to typical and atypical brain functions (Canolty and Knight 2010). An example of *inter-frequency coupling* is phase-to-amplitude coupling (PAC), which is decisively involved in neural integration (Munia and Aviyente 2019).

Particularly, theta-gamma PAC facilitates information transfer in the entorhinal-hippocampal network during encoding and retrieval (Colgin 2015) and predicts WM performance, as with slower theta cycles allowing more gamma cycles, each representing an item to retain (Axmacher et al. 2010; Lisman and Idiart 1995; Sauseng et al. 2010, 2019). In MCI and AD patients, decreased gamma frequency and weaker theta-gamma PAC are linked to impaired memory performances (Goodman et al. 2018).

Resuming, while previous literature has identified correlations between rsEEG power spectrum and memory performance and decline, the ability of rsEEG features to predict memory performance in healthy adults remains unclear. The present study investigates how rsEEG patterns — specifically spectral density across different frequency bands and theta-gamma PAC — can predict memory performance in young adults. Using both verbal and visual stimuli, this investigation will focus on how rsEEG can predict WM, IR, and DR, encompassing both STM and LTM. This comprehensive approach will provide deeper insights into the relationship between resting-state neural dynamics and memory processes, aiming to clarify the connection between baseline neural activity and memory performance, with potential implications for the early detection of memory issues and a broader understanding of brain oscillatory dynamics in cognitive functioning.

## Materials and methods

### Participants and procedure

Twenty-four young adults (13 F, *M*_age_ = 23.42, *SD*_age_ = 1.86, *M*_edu_*=* 16.62, *SD*_edu_*=* 1.74) without any history of neurological or psychiatric conditions were recruited from local universities and communities. Participants first provided informed consent and completed the Italian version of the Edinburgh Handedness Inventory (Oldfield 1971; Salmaso and Longoni 1985). They also completed the online short version of the Cognitive Reserve Index questionnaire (Mondini et al. 2023). Participants were selected to minimize variability in factors known to influence memory performance, such as age, education, and CRI scores. As a result, the sample was homogeneous in terms of age, sex distribution, and CRI scores (Table 1).

**Table 1:** Descriptive data of recruited participants. CRI = cognitive reserve index

	*N*	Mean	SD
Sex	13 F, 11 M		
Handedness	22 Dx, 2 Sn		
Age (in years)	24	23,42	1,86
Education (in years)	24	16,25	1,74
CRI (Tot)	24	98,75	5,30
CRI_Education	24	106,00	11,31
CRI_Working Activity	24	92,17	2,56
CRI_Leisure Time	24	98,00	4,04

**Fig. 1:**
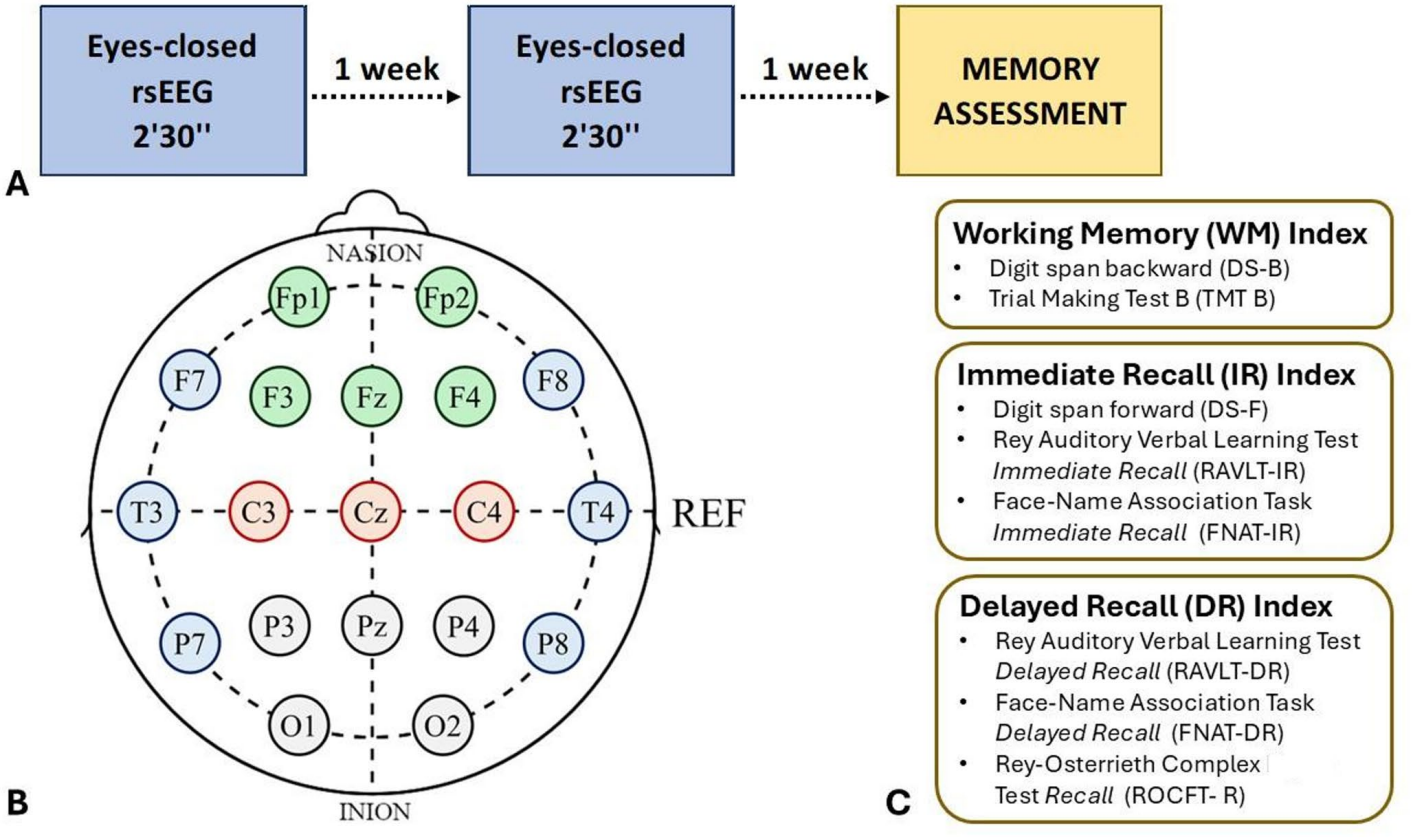
(**A**) Experimental protocol. (**B**) Electroencephalography (EEG) montage and Regions of Interest (ROI): Green electrodes represent the frontal ROI, Red electrodes represent the central ROI, Blue electrodes represent the temporal ROI, and gray electrodes represent the posterior ROI. (**C**) Memory indices were calculated by averaging the z-scores of specific (sub) test scores that assessed distinct memory processes: working memory, immediate recall, and delayed recall

The study adhered to the ethical standards of the World Medical Association Declaration of Helsinki, and the protocol received approval from the Ethics Committee of the University of Bari Aldo Moro (protocol number: ET-24–01). The experiment consisted of two eyes-closed rsEEG sessions of 2 min and 30 s each, conducted one week apart at the same time of day. This design was chosen to account for potential intra-individual variability in rsEEG over time, ensuring a more stable measurement of neural activity. To investigate correlations between baseline neural activity and subsequent cognitive performance without immediate test-related influences and avoid potential fatigue-related effects on cognitive performance, the memory assessment was scheduled one week after the second rsEEG session (see Fig. 1A).

### Neuropsychological assessment

The battery administered to each participant consisted of five memory tests:

(i) the Digit Span (DS; Spinnler and Tognoni 1987), comprising two subtests: the Digit Span Forward (DS-F) and the Digit Span Backward (DS-B). The DS-F evaluates auditory immediate recall by requiring participants to listen to and repeat increasingly long sequences of numbers (up to 9 digits, two sequences for each length). The DS-B assesses verbal WM by asking participants to repeat the same sequences of numbers in reverse order. Both subtests were administered via computer and scored by the experimenter through the total number of correctly recalled sequences.
(ii) the A and B forms of the Trail Making Test (TMT; Giovagnoli et al. 1996), respectively measuring visuospatial attention and distributed attention and/or visuospatial WM (e.g., Pérez-Parra and Restrepo-de-Mejía, 2023). This task requires them to maintain and manipulate spatial information while shifting attention between different sets of stimuli.
(iii) the Face-Name Association Task (FNAT; Manippa et al. 2025) measures associative memory by requiring participants to learn and recall associations between faces and names. It consists of two subtests: the first assessing cross-modal memory (i.e., face-name associations) immediately after the encoding phase and the second examining the associations after a 15-minute delay.
(iv) the Rey Auditory Verbal Learning Test (RAVLT; Caltagirone et al. 1995) assesses verbal memory abilities by presenting participants with a list of 15 words for 5 consecutive presentations. Immediate memory is assessed by asking participants to recall as many words as possible after each presentation. The test also includes a free delayed recall trial, where participants are asked to recall the words after a 15-minute delay.
(v) the Rey-Osterrieth Complex Figure Test (ROCFT; Caffarra et al. 2002) assesses visuospatial constructional ability and visuo-spatial memory. Participants are asked to copy a complex geometric figure and later recall and draw it from memory, providing a measure of delayed visuospatial recall.

The entire battery lasted about 60 min. Raw scores from each (sub) test were converted into z-scores using the current sample data and averaged based on the memory process evaluated by the specific (sub) tests (see Fig. 1C). Specifically, the z-scores of the DS-B and TMT-B were averaged to obtain a WM index. The z-scores of the DS-F, RAVLT immediate recall, and FNAT immediate recall were averaged to obtain an Immediate Recall (IR) index. The z-scores of the RAVLT free delayed recall, FNAT free delayed recall, and ROCFT free delayed recall were averaged to obtain a Delayed Recall (DR) index. The higher (> 0) the z-score to the corresponding index, the better the participant’s performance.

### rsEEG data recording processing

EEG was recorded via a battery-driven portable 19-channel Starstim 20/32 system (Neuroelectrics, Spain) at a sampling rate of 500 Hz. The neoprene head cap was fitted with 19 electrodes according to the international 10–20 system and electrically referenced to a clip electrode placed in the right ear lobe (see Fig. 1B). Electrode impedances were kept below 10 kΩ throughout the recording. EEG was acquired for 150 s by the Starstim software suite (NIC) and analyzed offline with the EEGlab toolbox in Matlab 2023 (MathWorks Inc., USA).

EEG data was first filtered using a high-pass filter of 1 Hz and a low-pass filter of 150 Hz. A notch filter was set at 50 Hz. Recordings were referenced to the common average of all electrodes and Artifact Subspace Reconstruction algorithm (Chang et al., 2018) EEGlab plugin was performed to identify and correct or remove segments exceeding predefined amplitude thresholds. Recordings were then visually inspected to exclude further artifacts that survived. After artifact rejection, an average of 105.64s (*SD* = 35.63) of clean EEG data per session was retained. Then, independent component analysis (ICA), applied to the continuous data, was used to decompose the measured EEG signals into independent components. Those associated with artifacts (i.e., Muscle, Eye, Heart, and Channel Noise) were identified and removed (ranges 0–4, Mdn = 3 per trace).

The spectral segmentation of the EEG data was then conducted using Brainstorm toolbox for Matlab (Tadel et al. 2011). Welch’s method was applied by averaging 1s signal windows, with 50% overlap calculated using the Fast Fourier Transform (FFT) algorithm. Then, power spectral density (PSD) or periodogram was computed in Signal units^2^/Hz for each electrode in five frequency bands (i.e., Theta (θ) 4–7 Hz; Alpha (α) = 8–13 Hz; Beta (β) = 14–29 Hz, low-Gamma (l-γ) = 30–49 Hz; high-Gamma (h-γ); 51–100 Hz). Theta-Gamma phase-amplitude coupling (PAC) was computed through Brainstorm’s PAC toolbox, using theta band (4–7 Hz) as the low-frequency phase signal, while the entire gamma band (30–100 Hz) was operated as the high-frequency amplitude signal. The coupling was assessed by calculating the modulation index (MI), which measures the strength of PAC between the theta phase and gamma amplitude, allowing for the identification of cross-frequency interactions in the EEG data. Finally, for both PSD and theta-gamma PAC MI, EEG electrodes were clustered into four Region of Interests (ROIs; see Fig. 1B) by averaging the activity of the respective electrodes: (i) Frontal (Fp1, Fp2, F3, F4, Fz); (ii) Central (C3, C4, Cz); (iii) Temporal (F7, F8, T7, T8, P7, P8); and (iv) Posterior (P3, P4, Pz, O1, O2).

### Data analysis

The first step in the data analysis was to compute a correlation matrix to examine the relationships among WM, IR, and DR indices. This matrix allowed to identify the strength and direction of associations between memory measures, providing an overview of how these cognitive functions are interrelated. To control for multiple comparisons, the significance threshold was adjusted using the Benjamini-Hochberg false discovery rate (FDR) correction. Subsequently, for each memory measure (WM, IR, DR), five separate simple linear regression models were established, corresponding to the different EEG frequency bands: θ, α, β, l-γ, and h-γ. The band power within each ROI—Frontal, Central, Temporal, and Posterior—served as predictors in each model using the enter method, meaning that all predictor variables were included simultaneously without an automatic selection process. Three further linear regression models were conducted using memory measures and Theta-Gamma PAC MI within each ROI as predictors. Potential covariates such as age, education, and CRI scores, well known to influence memory performance, were not included in the regression models, being controlled by ensuring a homogeneous sample.

To assess and manage potential multicollinearity among predictors, tolerance and variance inflation factors (VIFs) were computed for each significant model. Predictors with high VIF values, indicating excessive collinearity, were examined further, and if necessary, adjustments were made, such as removing highly redundant predictors or applying dimensionality reduction techniques. Considering the small sample size and the exploratory aim of the study, no corrections for multiple comparisons were applied. Nonetheless, to enhance the reliability of significant models, we applied bootstrapping with 5,000 resamples.

## Results

**Fig. 2:**
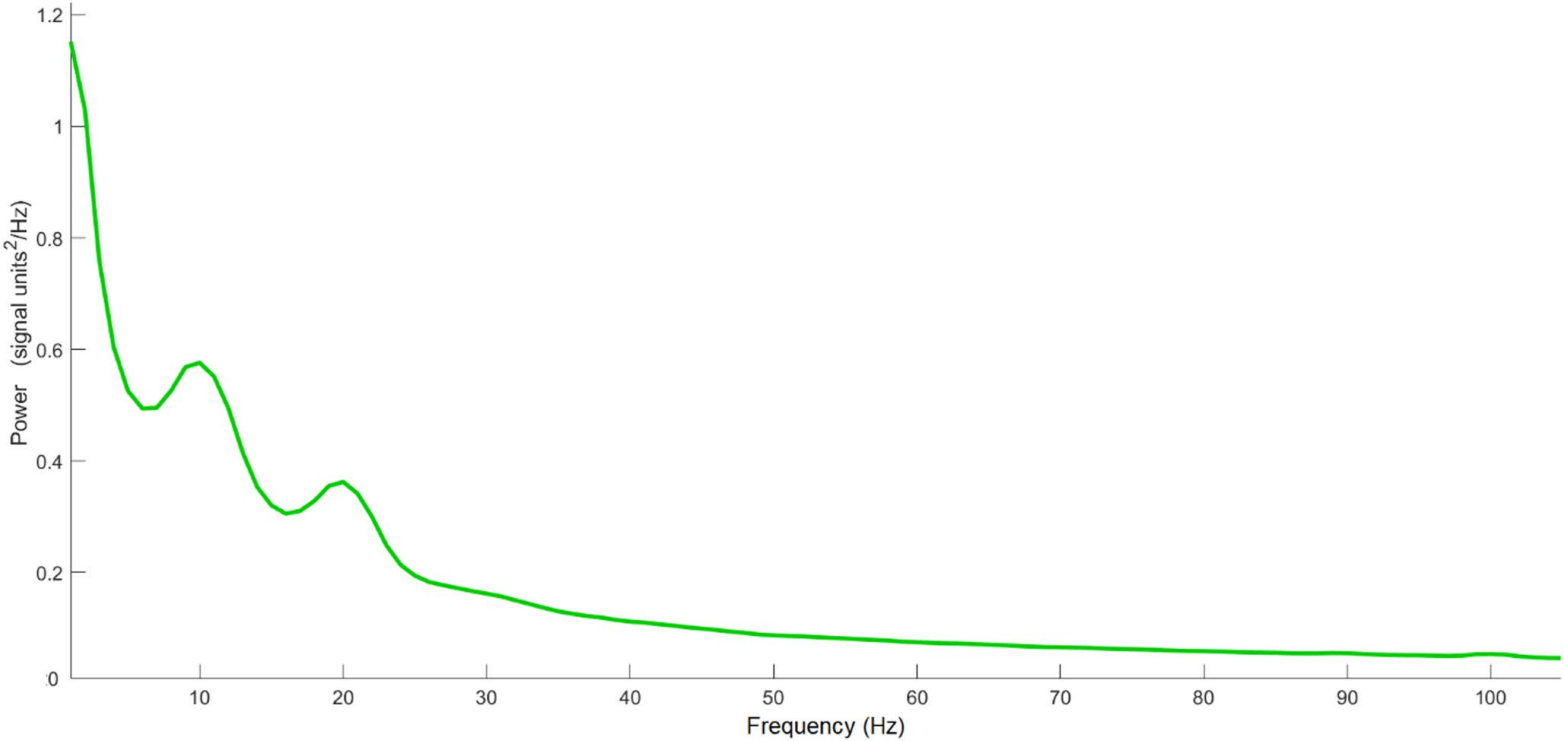
Average resting-state EEG (rsEEG) power spectral density (PSD) across all participants, recorded using a 19-channel Starstim 20/32 system. The mean spectrum shows a prominent peak around 10 Hz within the alpha band, reflecting typical resting-state alpha activity, and a secondary peak around 20 Hz within the beta band, which may relate to sensorimotor and cognitive idling processes during rest. Beyond these peaks, the spectrum displays the expected slow-to-fast like decline in power across higher frequencies, including the beta and gamma ranges

Descriptive data of the test raw scores, band powers, and theta-gamma PAC MI within each ROI are reported in Supplementary Materials 1. The mean rsEEG spectrum is reported in Fig. 2.

The correlations matrix revealed a significant positive relationship between IR and DR scores (*r* =.849, *p* <.001, corrected *p* =.001) and any other significant correlation (see Table 2).

**Table 2:** Pearson’s matrix correlation between memory indices. Only DR and IR z-scores showed significant positive correlation

Variable		WM z-score	IR z-score	DR z-score
WM z-score	Pearson’s r	—		
	*p*-value correct *p*-value	— —		
IR z-score	Pearson’s r	0.267	—	
	*p*-value correct *p*-value	0.208 0.312	— —	
DR z-score	Pearson’s r	0.101	0.782	—
	*p*-value correct *p*-value	0.640 0.640	< 0.001 0.001	— —

**Fig. 3:**
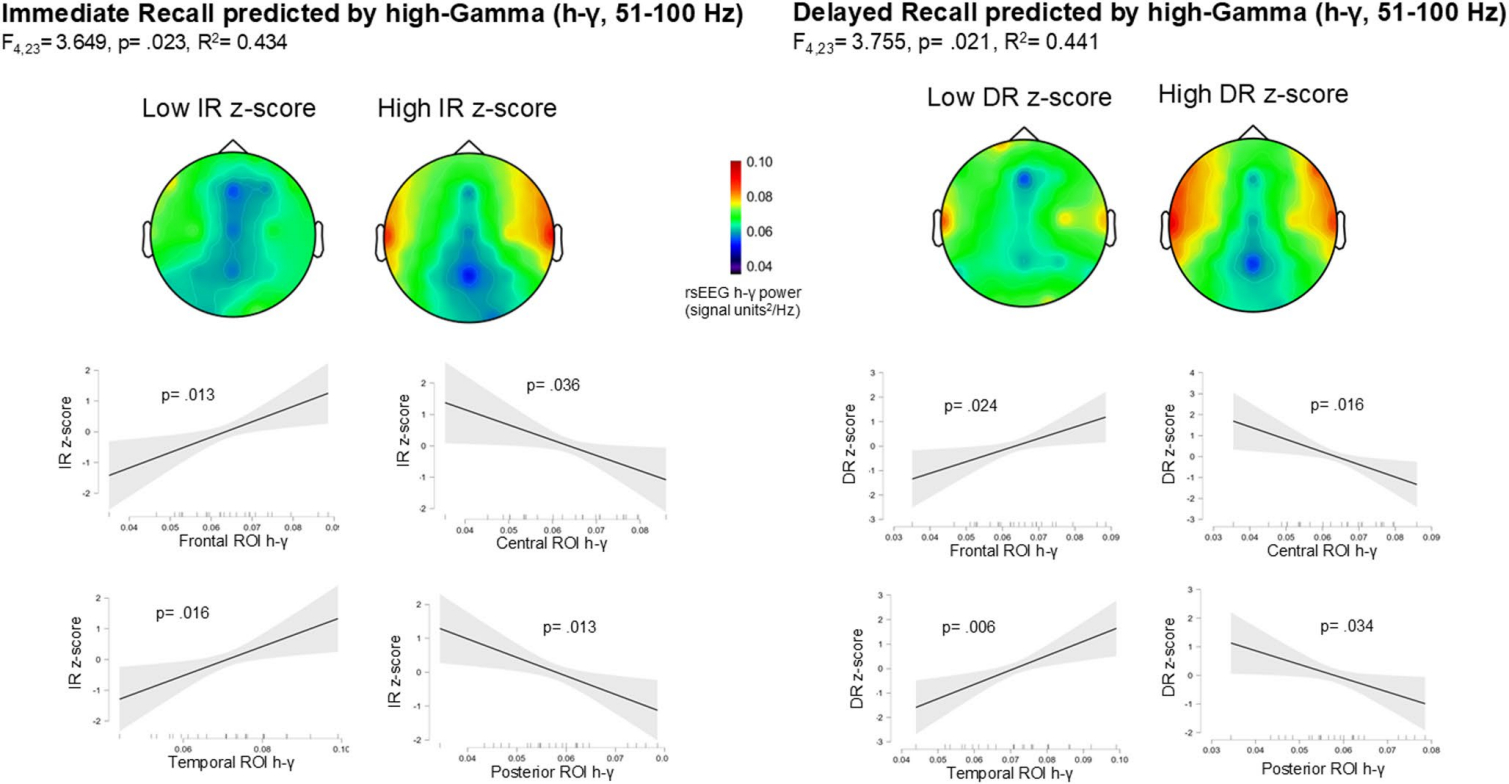
The figure illustrates the results of linear regression models predicting Immediate Recall (IR; left) and Delayed Recall (DR; right) based on resting-state high-Gamma (h-γ) power. Each topographic plot represents the averaged rsEEG activity of the four highest-performing and four lowest-performing individuals (2 males and 2 females) for display purposes only. Below the topoplot, scatterplots display the marginal effects of h-γ power across four regions of interest (ROI) and memory z-scores, with shaded areas indicating 95% confidence intervals for marginal means. Statistical significance levels (*p*-values) are provided for each association

The linear regression models to assess whether rsEEG power (Signal units^2^/Hz) within the four different ROIs predicts memory measures (i.e., WM, IR, and DR indices) yielded two statistically significant results (Fig. 3): (i) The regression model using IR Index as DV and ROIs’ h-γ power as predictors was statistically significant (R²= 0.434, adjusted R²= 0.315, RMSE = 0.539, F_(4,23)_ = 3.649, *p* =.023). Particularly, Frontal and Temporal h-γ showed a significant positive association with IR performance (β = 0.946, *p* =.013; β = 0.980, *p* =.016, respectively), while Posterior and Central h-γ showed a negative association (β= -2.263, *p* =.036; β= -2.724, *p* =.013, respectively). The VIF values ranged from 3.620 (Posterior h-γ) to 6.153 (Central h-γ), with Central h-γ showing the highest collinearity (VIF = 6.153, Tolerance = 0.163). Although VIF values above 5 can indicate moderate to high multicollinearity, no variables exceeded the commonly used threshold of 10, suggesting that collinearity was present but within acceptable limits. The bootstrapped confidence intervals (95% CI) confirmed the significance of the predictors, with Frontal h-γ (b = 49.504, *p* =.003, 95% CI [16.263, 103.425]) and Temporal h-γ (b = 48.864, *p* =.044, 95% CI [2.434, 89.897]) showing positive associations with IR performance. Conversely, Central h-γ (b= -48.416, *p* =.020, CI [-107.850, -9.045]) and Posterior h-γ (b= -53.036, *p* =.004, CI [-101.558, -19.262]) retained their negative associations.

(ii) The regression model using DR Index as DV and ROIs’ h-γ power as predictors was statistically significant (R²= 0.441, adjusted R²= 0.324, RMSE = 0.566, F_(4,23)_ = 3.755, *p* =.021). Particularly, Frontal and Temporal h-γ showed a significant positive association with DR performance (β = 0.847, *p* =.024, β = 1.144; *p* =.006, respectively), while Central and Posterior h-γ showed a negative association (β =-1.127, *p* =.016; β= -0.744, *p* =.034, respectively). The VIF values ranged from 3.620 (Posterior h-γ) to 6.153 (Central h-γ), with Central h-γ presenting the highest collinearity (VIF = 6.153, Tolerance = 0.163). Although values above 5 may indicate moderate to high multicollinearity, none of the predictors exceeded the critical threshold of 10, suggesting that collinearity was present but remained within acceptable limits. The bootstrapped confidence intervals confirmed the stability of the estimates: Frontal and Temporal h-γ remained positively associated with DR performance (b = 48.422, *p* =.025, CI [5.638, 95.404]; b = 59.281, *p* =.018, CI [19.089, 90.073], respectively), while Central and Posterior h-γ showed consistent negative associations (b= -59.032, *p* =.008, CI [-117.372, -30.551]; b= -47.415, *p* =.028, CI [-90.182, -7.915], respectively).

**Fig. 4:**
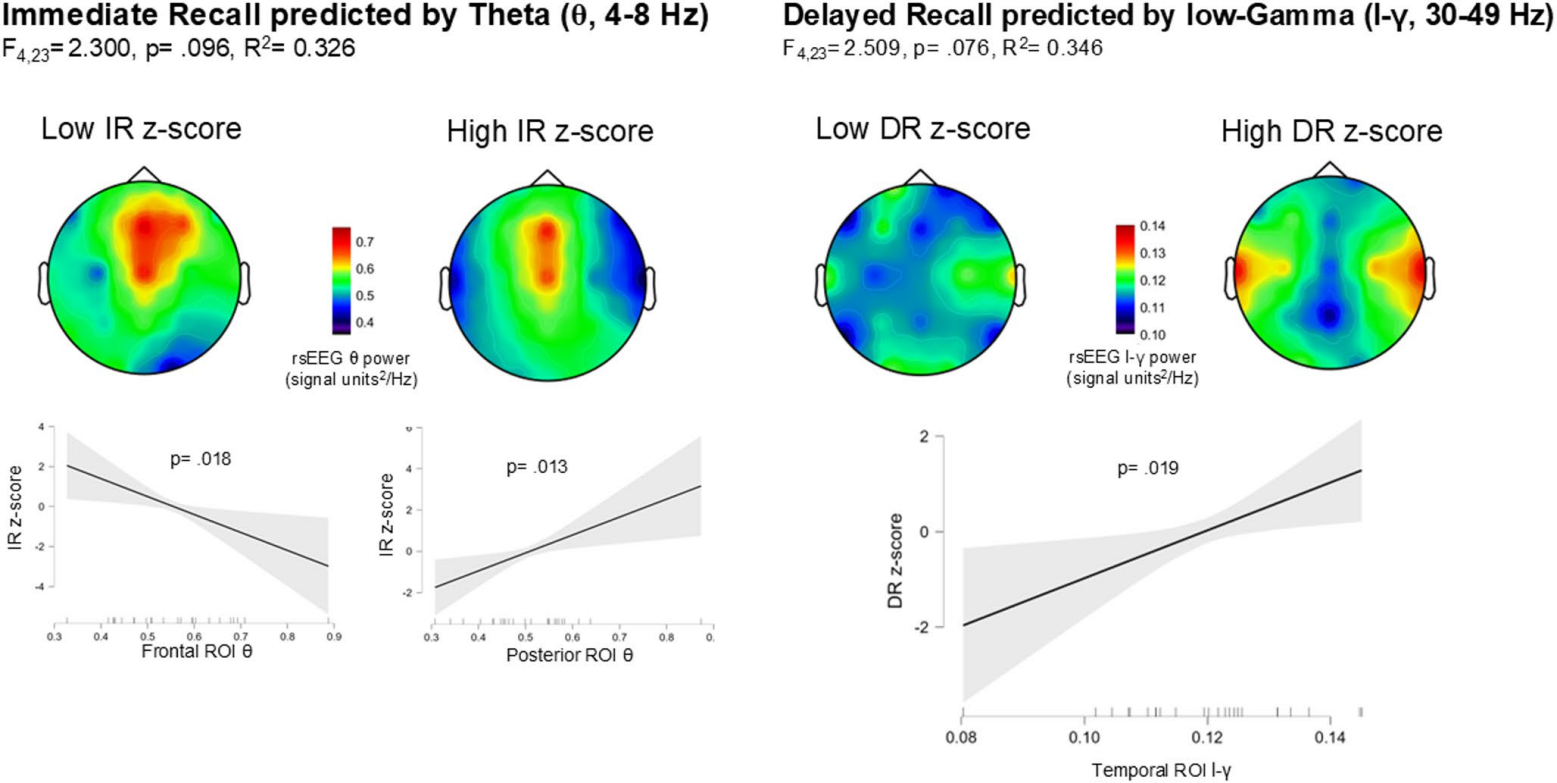
This figure presents the results of linear regression models predicting Immediate Recall (IR) based on resting-state Theta (θ) power (left) and Delayed Recall (DR) based on resting-state high-Gamma (l-γ) power (right). Each topographic plot illustrates the averaged rsEEG activity of the four highest-performing and four lowest-performing individuals (2 males and 2 females) for display purposes only. Below, scatterplots display the significant marginal effects of Frontal and Posterior θ power on IR z-score and Temporal l-γ power on DR z-score, with shaded areas representing 95% confidence intervals for marginal means. Statistical significance levels (*p*-values) are provided for each association

Other regression models showed approaching significance between band power and DV, particularly (see Fig. 4): (i) The regression model using IR Index as DV and ROIs’ θ as predictors was almost significant (R²= 0.326, F_(4,23)_ = 2.300, *p* =.096). Posterior θ showed a significant positive association (β = 1.511, *p* =.013) while Frontal θ showed a negative association (β= -1.728, *p* =.018) with IR performance, respectively. (ii) The regression model using DR Index as DV and ROIs’ l-γ power as predictors was almost significant (R²= 0.346, F_(4,23)_ = 2.509, *p* =.076). Temporal l-γ showed a significant positive association (β = 1.056, *p* =.019) with DR performance.

All other models across the different EEG bands were not significant. However, although the regression model using DR Index as DV and ROIs’ α power as predictors was not statistically significant (R²= 0.232, F_(4,23)_ = 1.434, *p* =.261), Temporal α (β= -0.899, *p* =.044) and Central α (β = 1.008, *p* =.045) were found to negatively and positively predict DR, respectively. Similarly, although the regression model using WM Index as DV and ROIs’ β power as predictors was not significant (R²= 0.307, F_(4,23)_ = 2.103, *p* =.120), WM was negatively predicted by Temporal β power (β= -1.524, *p* =.035). None of the regression models assessing whether rsEEG Theta-Gamma PAC MI predicts memory measures (i.e., WM, IR, and DR indices) reached statistical significance. However, although the regression model using the DR Index as DV and ROIs’ Theta-Gamma PAC MI as predictors was not significant (R²= 0.227, F_(4,23)_ = 1.393, *p* =.274), Posterior MI was negatively associated with DR performance (β= -1.158, *p* =.033). All statistical outputs of the regression models are reported in Supplementary Materials 2 and 3.

## Discussion

This study investigated how resting-state EEG (rsEEG) features, measured during two separate eyes-closed recording sessions, relate to memory performance in healthy young adults. Specifically, EEG power in various frequency bands and theta-gamma phase-amplitude coupling (PAC) were examined. By using multiple verbal and non-verbal neuropsychological tests, three memory processes were assessed: working memory (WM), immediate recall (IR), and delayed recall (DR), involved in both short-term (STM) and long-term memory (LTM) storage. The analysis revealed that IR and DR performances were strongly correlated and predicted by rsEEG gamma (γ) activity. Other interesting results involved the role of theta activity in IR performance. These results emphasize the distinct roles that specific rsEEG bands play in different aspects of memory function.

The key finding is the association between spontaneous γ power and memory performances, with high gamma (h-γ, 51–100 Hz) activity accounting for 43% and 44% of the IR and DR indices variance, respectively, with adjusted R² values of 0.32 for both indices, suggesting that the models explain a moderate portion of the variance even after accounting for the number of predictors. Moreover, the models’ Root Mean Square Error (RMSE) values of about 0.55 indicate that h-γ provides reasonably accurate predictions of memory performances. IR and DR depend on two critical memory processes: encoding information with focused attention and later retrieving this learned information. Encoding, which relies on sustained attention, ensures successful storage in memory, while efficient delayed recall depends on accessing stored information over time, making accurate retrieval impossible without a successful attentive encoding process. Accordingly, IR and DR performance correlated, associating positively with frontal and temporal h-γ and negatively with central and posterior h-γ. These associations were confirmed after bootstrapping with 5,000 resamples. Additionally, the multicollinearity among the predictors was found to be tolerable, further ensuring the robustness of the predictions and the stability of the model.

The role of γ for LTM processes, particularly in episodic memory, has been extensively demonstrated in literature, primarily within the medial temporal and prefrontal cortices (Griffiths and Jensen 2023). Coherent with findings, hippocampal h-γ activity is linked to neural coordination within memory-related networks, contributing to LTM encoding and storage (Kucewicz et al. 2017; Nyhus and Curran 2010). Similarly, more sustained spontaneous prefrontal h-γ activity may facilitate executive functions that support memory retrieval. Studies on cognitive control suggest that the frontal region is involved in organizing and retrieving information, especially for tasks requiring higher cognitive effort (Cho et al. 2006; Roux and Uhlhaas 2014). Thus, frontal γ activity may help reinforce memory networks, aiding the retrieval of previously encoded information and supporting both IR and DR. Neuromodulation studies in healthy participants support this hypothesis, showing that the most effective LTM enhancement is achieved through 60 Hz transcranial alternating current stimulation (tACS) targeting the prefrontal cortex (Booth et al. 2022; Grover et al. 2022; Manippa et al. 2024b). Congruent with this evidence, the observed results suggest that a spontaneous sustained h-γ activity during rest might indicate a readiness or priming of memory-related networks, leading to efficient encoding, storage, and retrieval processes. This sustained γactivity may serve as a foundational mechanism, supporting IR and DR by maintaining neural excitability and connectivity across temporo-frontal pathways.

In contrast, a negative association between IR and DR performance and spontaneous h-γ power in the central and posterior ROIs was found. Compared to temporal and frontal areas, increased resting state h-γ activity in these ROIs may indicate heightened sensory/motor readiness. This bias towards sensory/motor integration could potentially limit higher-order processing more represented in temporo-frontal cortices. As a result, cognitive resources required for effective encoding may be reduced, potentially explaining difficulties in IR performance (Lega et al. 2012). Similarly, high resting h-γ activity in the central and posterior ROIs hinders DR. This observation is consistent with research suggesting that spontaneous posterior γ activity can disrupt memory consolidation and retrieval processes, particularly when the brain prioritizes sensorimotor over integrative functions (Hanslmayr et al. 2016). Consequently, elevated posterior h-γ power may interfere with LTM acquisition and retention, impairing the ability to access learned information during delayed recall. This pattern aligns with the possibility that a sensory-dominant state, characterized by high posterior γ activity, might compete with or inhibit the neural processes essential for memory consolidation.

Other marginal associations emerged between rsEEG l-γ power and DR and between θ and IR. The positive significant association between spontaneous temporal l-γ (30–49 Hz) and DR performance is consistent with growing evidence highlighting the critical role of l-γ particularly for hippocampal long-term potentiation (LTP) processes, the neural substrates of both encoding and recall processes of LTM (Dvorak et al. 2018; Trimper et al. 2017). Congruently, temporal l-γ (40 Hz) tACS is one of the most promising neuromodulatory approaches under investigation for MCI and AD (Manippa et al. 2024a), as early stages of both conditions are characterized by rsEEG γ slowing and reduction (e.g., Güntekin et al. 2023), and temporal atrophy (e.g., Scianatico et al. 2024). These findings underscore the importance of spontaneous temporal l-γ activity for effective DR, likely supporting hippocampal LTP and related memory processes. On the other hand, resting-state posterior θ power positively predicts IR abilities, with frontal θ negatively associated. This dual pattern of θ power reflects the distinct functions of different brain regions: frontal θ may represent cognitive control processes that are less beneficial for the rapid encoding demands, while spontaneous posterior θ activity involved in attentional processes (e.g. Asanowicz et al. 2023; Yang et al. 2017), may support information encoding. Accordingly, Osipova et al. (2006) highlighted that θ activity over right parietotemporal areas is directly engaged in mnemonic processes during declarative memory tasks. Furthermore, extended literature suggests the positive effects of parietal θ neurostimulation on STM performances, involving both IR and WM (Booth et al. 2022; Grover et al. 2022). These findings reveal a complex yet consistent pattern: spontaneous γ activity, primarily in the temporal and frontal cortices, positively correlates with multimodal IR and DR performance. Additionally, spontaneous θ activity appears to predict IR performance. These results align with current literature, highlighting the roles of spontaneous θ and γ activity in predicting memory processes in healthy individuals (e.g., Nyhus and Curran 2010; Osipova et al. 2006). Furthermore, they underscore how disruptions or alterations in these activities are implicated in neurodegenerative processes that primarily impair memory performance (e.g., Moretti et al. 2009; Şeker and Özerdem 2024).

Albeit no other significant model was identified, some predictors might influence memory processes, warranting further investigation. Importantly, these individual β coefficients emerged within the context of overall non-significant models, and thus should be interpreted with caution. For instance, in the non-significant α-DR model, central and temporal α activity exhibited an opposing association with DR scores. Notably, previous research has highlighted the critical role of α activity in memory performance (e.g., Amin et al. 2025; Anderson and Perone 2018; Luckhaus et al. 2008). Alpha rhythms are generally linked to attentional disengagement and semantic memory processes (Klimesch 1999). The absence of significant models in the present study may reflect the limited variability and predictive power of α activity in young adults, who typically possess well-established attentional and cognitive control mechanisms, particularly when not actively engaged in a task. Furthermore, although the literature suggests a potential relationship between specific rsEEG activity and WM tasks (Kucewicz et al. 2017; Mably and Colgin 2018), findings did not support this hypothesis. A negative association between rsEEG β power and WM was observed, yet this was part of an overall non-significant model, and thus must be interpreted cautiously. While WM may not be directly associated with the resting-state spectral features we examined, this does not rule out that task-related EEG patterns, such as event-related potentials (ERPs) or task-induced spectrum, could offer more precise insights into WM performance. The absence of a clear link between resting-state spectrum features and WM performance may reflect the complexity and multidimensional nature of WM, consistent with the lack of correlation between WM and IR or DR indices. While WM is considered a component of STM, it relies on higher cognitive processes, such as executive functions, that may not be fully captured by rsEEG (Friedman and Miyake 2004). In this regard, task-related activity may better reflect cognitive functions requiring active manipulation of information—processes that are more central to WM tasks—than the spontaneous activity observed during rest (Moran et al. 2010). Studies adopting these paradigms have found strong evidence between EEG features (notably θ and α activity, as well as theta-gamma PAC) and WM performance (Sauseng et al. 2010; Moran et al. 2010).

Similarly, although the role of theta-gamma PAC in memory processes is well-established, findings show that spontaneous PAC did not predict memory performance, with the only exception being posterior theta-gamma PAC, which showed a negative association with DR scores within a non-significant model. As with other isolated associations, this result should be interpreted cautiously, given that it did not emerge from a statistically significant overall model. This outcome is not unexpected, considering resting-state activity primarily reflects baseline brain dynamics, whereas cross-frequency coupling is typically observed during task engagement. Particularly, theta-gamma PAC is supposed to facilitate memory encoding and retrieval (Lisman and Idiart 1995; Sauseng et al. 2010), supporting information transfer between the hippocampus and prefrontal cortex (Colgin 2015; Lisman and Idiart 1995). As indicated by descriptive data, mean MI was very low, suggesting that spontaneous PAC is not relevant for predicting memory performance.

### Future directions, limitations, and conclusions

This study highlights that spontaneous γ activity in specific brain regions supports multimodal memory processes. Specifically, frontotemporal h-γ activity is positively associated with IR and DR, whereas central and posterior h-γ power during rest negatively predicts performance in these tasks. Additionally, IR is supported by posterior θ power but hindered by frontal θ activity, while DR performance is positively predicted by spontaneous temporal l-γ power. Future studies could investigate how resting state γ activity influences cognitive performance, particularly in tasks that require flexible retrieval and integration of episodic information, in which γ is strongly involved (Manippa et al. 2024c).

The present study is not without limitations. Although results align with prior research on γ oscillations and long-term memory (LTM), larger sample sizes and high-definition EEG would enable more nuanced analyses of individual differences and a deeper exploration of the role of different oscillation sources. Future research should aim to replicate these preliminary findings in larger and more diverse populations to enhance their robustness and applicability. Furthermore, expanding this research to older populations and those at risk for cognitive decline would provide valuable insights on age-related changes in oscillatory dynamics and their relationship to memory. Longitudinal studies may also facilitate investigation of resting-state γ activity as a preclinical biomarker for cognitive decline or as a tool for identifying early neurodegenerative processes. This is particularly relevant given the numerous studies demonstrating γ and memory disruption in AD and MCI patients (Başar 2013; Güntekin et al. 2023). Another limitation is our reliance on a specific set of memory tests, which may not fully capture the complexity of memory systems. While being among the first to demonstrate a strong relationship between rsEEG γ oscillations and multimodal LTM, it is also important to note that different cognitive tasks may engage WM components in distinct ways. For example, n-back tasks (the most commonly used task to assess WM) recruit multiple WM components and provide a more nuanced assessment compared to the digit span backward or trail-making tests used in the current study (Jaeggi et al. 2008; Zanto et al. 2011).

In conclusion, this study offers novel insights into the role of rsEEG spectral power and theta-gamma PAC in memory performance among young adults, particularly emphasizing the role of γ spontaneous activity. Findings underscore the importance of spontaneous fast-frequency oscillations in frontal and temporal regions in supporting memory processes, particularly IR and DR. These results add to the growing body of literature highlighting the role of oscillatory dynamics in cognitive functions and provide a foundation for future investigations of the neural mechanisms underlying memory performance across different age groups and clinical populations. Expanding upon this research with larger sample sizes, more comprehensive memory assessments, and refined cross-frequency analyses will further elucidate the complexities of brain oscillations and their relationship to human memory. Based on these findings, future studies could explore whether targeted interventions, such as neuromodulation techniques, might help recalibrate γ activity in these regions to enhance memory performance.

## Electronic Supplementary Material

Below is the link to the electronic supplementary material.

Supplementary Material 1

Supplementary Material 2

Supplementary Material 3

## Data Availability

The data supporting the results of this study are not publicly available due to privacy and participant protection considerations. However, data can be requested from the corresponding author (Gaetano Scianatico, gaetano.scianatico@uniba.it) and access will be granted upon approval, in accordance with applicable ethical and legal standards.
