# Supplementary material for: Resting-state EEG gamma power predicts immediate and delayed recall in healthy adults
Source: Cogn Neurodyn. 2025 Aug 26;19(1):138. doi: 10.1007/s11571-025-10313-2 (PMC12381311; doi:10.1007/s11571-025-10313-2)
Supplement: Supplementary file 1 — Supplementary Material 1 [file 11571_2025_10313_MOESM1_ESM.docx]

|  | Median | Mean | SD | IQR |
| --- | --- | --- | --- | --- |
| DS-B (Digit Span backward | 8 | 8.181 | 2.498 | 2 |
| TMT B (Trial Making Test B) | 64 | 62.643 | 17.489 | 19.5 |
| DS-F (Digit Span forward) | 7.5 | 7.892 | 2.314 | 2 |
| RAVLT_IR | 58.625 | 56.215 | 11.572 | 7.75 |
| FNAT_IR | 6 | 6.976 | 4.163 | 6.25 |
| RAVLT_DR | 13 | 12.547 | 2.589 | 2 |
| FNAT_DR | 9.104 | 9.024 | 4.327 | 6.25 |
| ROCF_Recall | 28 | 25.894 | 7.447 | 5.25 |
|  |  |  |  |  |
| **Supplementary Table 1**. The table presents the median, mean, standard deviation (SD), and interquartile range (IQR) for the raw scores of each test and subtest. Abbreviations: RAVLT = Rey Auditory Verbal Learning Test, FNAT = Face-Name Association Task, ROCF = Rey-Osterrieth Complex Figure Test (IR = Immediate Recall, DR = Delayed Recall). The TMT-B score is reported in seconds, while for DS-B and DS-F, the values represent the number of spans correctly recalled out of 14, rather than the span length. Scores for TMT-A and the ROCF copy task are not reported, as they are not used in calculating the memory indices employed as dependent variables. | | | | |

|  | Median | Mean | SD | IQR |
| --- | --- | --- | --- | --- |
| Th_Frontal | 0.549 | 0.555 | 0.125 | 0.174 |
| Th_Central | 0.558 | 0.561 | 0.116 | 0.147 |
| Th_Temporal | 0.493 | 0.498 | 0.112 | 0.136 |
| Th_Posterior | 0.505 | 0.509 | 0.116 | 0.123 |
| Al_Frontal | 0.516 | 0.532 | 0.102 | 0.135 |
| Al_Central | 0.53 | 0.532 | 0.091 | 0.139 |
| Al_Temporal | 0.468 | 0.471 | 0.089 | 0.128 |
| Al_Posterior | 0.563 | 0.566 | 0.068 | 0.102 |
| Be_Frontal | 0.265 | 0.264 | 0.056 | 0.076 |
| Be_Central | 0.272 | 0.272 | 0.056 | 0.089 |
| Be_Temporal | 0.239 | 0.248 | 0.048 | 0.076 |
| Be_Posterior | 0.312 | 0.304 | 0.063 | 0.102 |
| lG_Frontal | 0.119 | 0.119 | 0.019 | 0.024 |
| lG_Central | 0.117 | 0.119 | 0.016 | 0.02 |
| lG_Temporal | 0.121 | 0.119 | 0.015 | 0.016 |
| lG_Posterior | 0.121 | 0.118 | 0.017 | 0.02 |
| hG_Frontal | 0.063 | 0.063 | 0.012 | 0.014 |
| hG_Central | 0.066 | 0.064 | 0.013 | 0.022 |
| hG_Temporal | 0.072 | 0.071 | 0.013 | 0.019 |
| hG_Posterior | 0.057 | 0.058 | 0.011 | 0.01 |

| **Supplementary Table 2**. The table presents the median, mean, standard deviation (SD), and interquartile range (IQR) for the rsEEG power (in Signal units^2^/Hz) of each frequency within each ROI. Th= Theta, Al= Alpha, Be= Beta, lG= low-Gamma, hG= high-Gamma |
| --- |

|  | Median | Mean | SD | IQR |
| --- | --- | --- | --- | --- |
| Theta-Gamma PAC_Frontal | 0.075 | 0.073 | 0.011 | 0.015 |
| Theta-Gamma PAC_Central | 0.072 | 0.074 | 0.012 | 0.008 |
| Theta-Gamma PAC_Temporal | 0.067 | 0.067 | 0.011 | 0.014 |
| Theta-Gamma PAC_Posterior | 0.068 | 0.070 | 0.012 | 0.013 |

**Supplementary Table 3**. The table presents the median, mean, standard deviation (SD), and interquartile range (IQR) for the rsEEG Theta-Gamma Phase-Amplitude Coupling (in Modulation Index) of each frequency within each ROI.


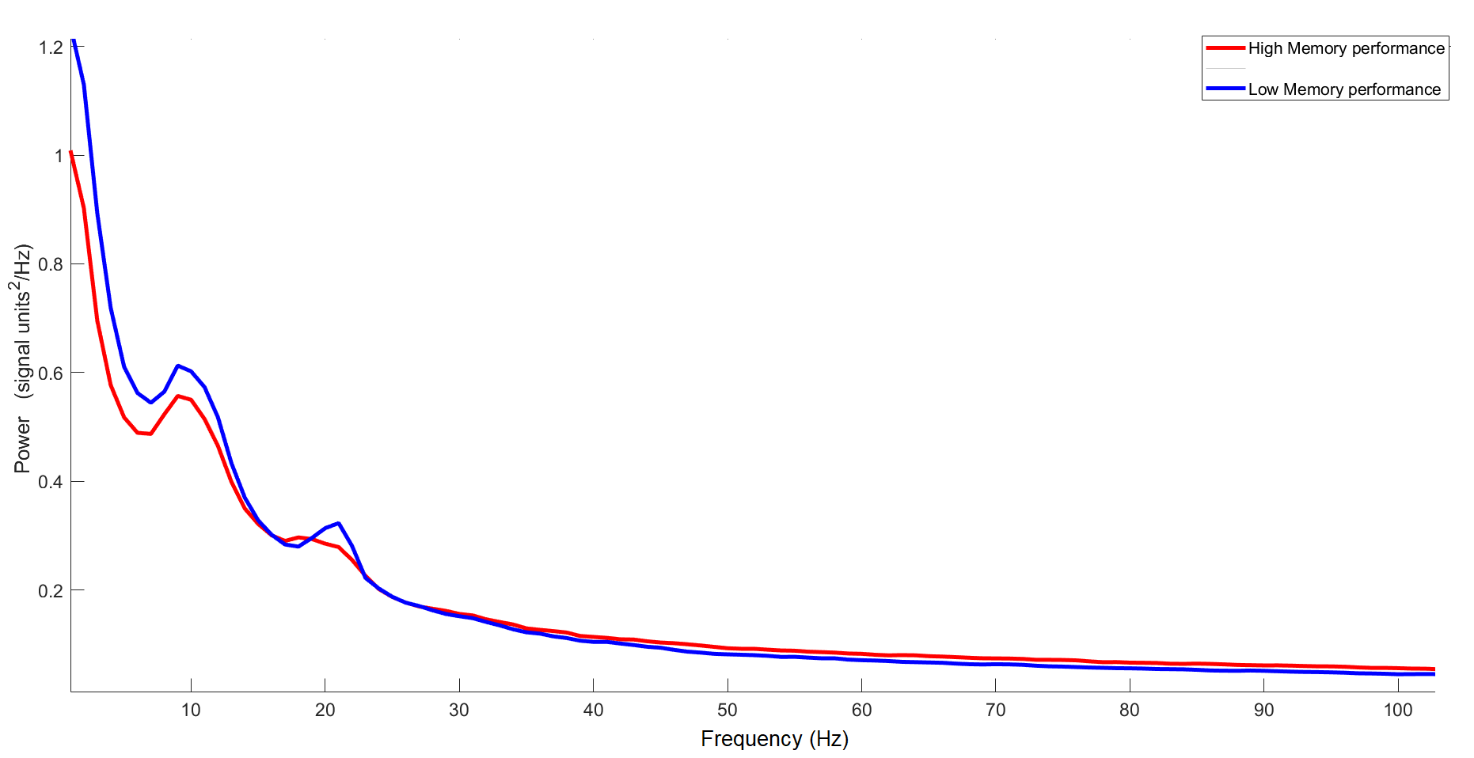


**Supplementary Figure 1.** The figure shows the resting-state EEG power spectra (1–100 Hz) averaged for the four highest-performing (red line) and four lowest-performing (blue line) individuals (2 males and 2 females per group) for display purposes only.
