# Supplementary material for: Resting-state EEG gamma power predicts immediate and delayed recall in healthy adults
Source: Cogn Neurodyn. 2025 Aug 26;19(1):138. doi: 10.1007/s11571-025-10313-2 (PMC12381311; doi:10.1007/s11571-025-10313-2)
Supplement: Supplementary file 2 — Supplementary Material 2 [file 11571_2025_10313_MOESM2_ESM.pdf]

Results

Linear Regression Th_WM

Model Summary - WM z-score

Model	R	R^2	Adjusted R^2	RMSE
M_0	0.000	0.000	0.000	0.752
M_1	0.226	0.051	−0.149	0.806

Note. M_1 includes Th_Frontal, Th_Central, Th_Temporal, Th_Posterior

ANOVA

Model		Sum of Squares	df	Mean Square	F	p
M_1	Regression	0.665	4	0.166	0.256	0.902
	Residual	12.355	19	0.650		
	Total	13.021	23			

Note. M_1 includes Th_Frontal, Th_Central, Th_Temporal, Th_Posterior
Note. The intercept model is omitted, as no meaningful information can be shown.

Coefficients

Model		Unstandardized	Standard Error	Standardized	t	p
M_0	(Intercept)	-2.266×10^{-17}	0.154		-1.476×10^{-16}	1.000
M_1	(Intercept)	−0.157	0.849		−0.185	0.855
	Th_Frontal	−2.236	4.733	−0.372	−0.472	0.642
	Th_Central	1.276	3.637	0.197	0.351	0.730
	Th_Temporal	−2.112	3.001	−0.314	−0.704	0.490
	Th_Posterior	3.409	4.341	0.526	0.785	0.442

Linear Regression Th_IR*

Model Summary - IR z-score

Model	R	R²	Adjusted R²	RMSE
M_0	0.000	0.000	0.000	0.651
M_1	0.571	0.326	0.184	0.588

Note. M_1 includes Th_Frontal, Th_Central, Th_Temporal, Th_Posterior

ANOVA

Model		Sum of Squares	df	Mean Square	F	p
M_1	Regression	3.178	4	0.794	2.300	0.096
	Residual	6.563	19	0.345		
	Total	9.740	23			

Note. M_1 includes Th_Frontal, Th_Central, Th_Temporal, Th_Posterior
Note. The intercept model is omitted, as no meaningful information can be shown.

Coefficients

Model		Unstandardized	Standard Error	Standardized	t	p
M_0	(Intercept)	-1.700×10^{-17}	0.133		-1.280×10^{-16}	1.000
M_1	(Intercept)	0.277	0.619		0.448	0.659
	Th_Frontal	−8.972	3.450	−1.728	−2.601	0.018
	Th_Central	3.179	2.651	0.568	1.199	0.245
	Th_Temporal	−3.020	2.187	−0.519	−1.381	0.183
	Th_Posterior	8.694	3.164	1.551	2.748	0.013

Linear Regression Th_DR

Model Summary - DR z-score

Model	R	R^2	Adjusted R^2	RMSE
H_0	0.000	0.000	0.000	0.688
H_1	0.475	0.226	0.063	0.666

ANOVA

Model		Sum of Squares	df	Mean Square	F	p
H_1	Regression	2.459	4	0.615	1.385	0.277
	Residual	8.433	19	0.444		
	Total	10.892	23			

Note. The intercept model is omitted, as no meaningful information can be shown.

Coefficients

Model		Unstandardized	Standard Error	Standardized	t	p
H_0	(Intercept)	-2.266×10^{-17}	0.140		-1.613×10^{-16}	1.000
H_1	(Intercept)	0.543	0.701		0.774	0.448
	Th_Frontal	−6.831	3.910	−1.244	−1.747	0.097
	Th_Central	4.054	3.005	0.684	1.349	0.193
	Th_Temporal	−3.854	2.479	−0.626	−1.554	0.137
	Th_Posterior	5.689	3.586	0.960	1.586	0.129

Linear Regression Al_WM

Model Summary - WM z-score

Model	R	R^2	Adjusted R^2	RMSE
H_0	0.000	0.000	0.000	0.752
H_1	0.401	0.161	−0.016	0.758

ANOVA

Model		Sum of Squares	df	Mean Square	F	p
H_1	Regression	2.097	4	0.524	0.912	0.477
	Residual	10.924	19	0.575		
	Total	13.021	23			

Note. The intercept model is omitted, as no meaningful information can be shown.

Coefficients

Model		Unstandardized	Standard Error	Standardized	t	p
H_0	(Intercept)	-2.266×10^{-17}	0.154		-1.476×10^{-16}	1.000
H_1	(Intercept)	0.164	1.357		0.121	0.905
	Al_Frontal	4.642	2.604	0.628	1.783	0.091
	Al_Central	−1.350	4.051	−0.163	−0.333	0.743
	Al_Temporal	−1.496	3.703	−0.176	−0.404	0.691
	Al_Posterior	−2.138	3.088	−0.192	−0.692	0.497

Linear Regression AI_IR

Model Summary - IR z-score

Model	R	R²	Adjusted R²	RMSE
H_0	0.000	0.000	0.000	0.651
H_1	0.475	0.226	0.062	0.630

ANOVA

Model		Sum of Squares	df	Mean Square	F	p
H_1	Regression	2.197	4	0.549	1.383	0.277
	Residual	7.544	19	0.397		
	Total	9.740	23			

Note. The intercept model is omitted, as no meaningful information can be shown.

Coefficients

Model		Unstandardized	Standard Error	Standardized	t	p
H_0	(Intercept)	-1.700×10^{-17}	0.133		-1.280×10^{-16}	1.000
H_1	(Intercept)	−1.404	1.128		−1.244	0.228
	AI_Frontal	−3.020	2.164	−0.472	−1.396	0.179
	AI_Central	5.062	3.367	0.708	1.503	0.149
	AI_Temporal	−4.655	3.077	−0.634	−1.513	0.147
	AI_Posterior	4.426	2.566	0.460	1.725	0.101

Linear Regression AI_DR

Model Summary - DR z-score

Model	R	R²	Adjusted R²	RMSE
H_0	0.000	0.000	0.000	0.688
H_1	0.482	0.232	0.070	0.664

ANOVA

Model		Sum of Squares	df	Mean Square	F	p
H_1	Regression	2.526	4	0.632	1.434	0.261
	Residual	8.366	19	0.440		
	Total	10.892	23			

Note. The intercept model is omitted, as no meaningful information can be shown.

Coefficients

Model		Unstandardized	Standard Error	Standardized	t	p
H_0	(Intercept)	-2.266×10^{-17}	0.140		-1.613×10^{-16}	1.000
H_1	(Intercept)	−0.923	1.188		−0.778	0.446
	AI_Frontal	−1.920	2.278	−0.284	−0.843	0.410
	AI_Central	7.618	3.545	1.008	2.149	0.045
	AI_Temporal	−6.980	3.240	−0.899	−2.154	0.044
	AI_Posterior	2.072	2.702	0.203	0.767	0.453

Linear Regression Be_WM

Model Summary - WM z-score

Model	R	R^2	Adjusted R^2	RMSE
H_0	0.000	0.000	0.000	0.752
H_1	0.554	0.307	0.161	0.689

ANOVA

Model		Sum of Squares	df	Mean Square	F	p
H_1	Regression	3.995	4	0.999	2.103	0.120
	Residual	9.025	19	0.475		
	Total	13.021	23			

Note. The intercept model is omitted, as no meaningful information can be shown.

Coefficients

Model		Unstandardized	Standard Error	Standardized	t	p
H_0	(Intercept)	-2.266×10^{-17}	0.154		-1.476×10^{-16}	1.000
H_1	(Intercept)	−0.496	0.758		−0.654	0.521
	Be_Frontal	12.779	7.696	0.955	1.661	0.113
	Be_Central	8.861	6.370	0.655	1.391	0.180
	Be_Temporal	−23.838	10.520	−1.524	−2.266	0.035
	Be_Posterior	2.063	7.796	0.173	0.265	0.794

Linear Regression Be_IR

Model Summary - IR z-score

Model	R	R^2	Adjusted R^2	RMSE
H_0	0.000	0.000	0.000	0.651
H_1	0.290	0.084	−0.109	0.685

ANOVA

Model		Sum of Squares	df	Mean Square	F	p
H_1	Regression	0.819	4	0.205	0.436	0.781
	Residual	8.921	19	0.470		
	Total	9.740	23			

Note. The intercept model is omitted, as no meaningful information can be shown.

Coefficients

Model		Unstandardized	Standard Error	Standardized	t	p
H_0	(Intercept)	-1.700×10^{-17}	0.133		-1.280×10^{-16}	1.000
H_1	(Intercept)	−0.345	0.754		−0.457	0.653
	Be_Frontal	−6.877	7.651	−0.594	−0.899	0.380
	Be_Central	2.509	6.333	0.214	0.396	0.696
	Be_Temporal	−5.141	10.459	−0.380	−0.492	0.629
	Be_Posterior	9.068	7.750	0.877	1.170	0.256

Linear Regression Be_DR

Model Summary - DR z-score

Model	R	R^2	Adjusted R^2	RMSE
H_0	0.000	0.000	0.000	0.688
H_1	0.276	0.076	−0.118	0.728

ANOVA

Model		Sum of Squares	df	Mean Square	F	p
H_1	Regression	0.830	4	0.207	0.392	0.812
	Residual	10.062	19	0.530		
	Total	10.892	23			

Note. The intercept model is omitted, as no meaningful information can be shown.

Coefficients

Model		Unstandardized	Standard Error	Standardized	t	p
H_0	(Intercept)	-2.266×10^{-17}	0.140		-1.613×10^{-16}	1.000
H_1	(Intercept)	−0.676	0.801		−0.844	0.409
	Be_Frontal	−4.130	8.126	−0.338	−0.508	0.617
	Be_Central	2.613	6.726	0.211	0.389	0.702
	Be_Temporal	−4.489	11.107	−0.314	−0.404	0.691
	Be_Posterior	7.143	8.231	0.653	0.868	0.396

Linear Regression IG_WM

Model Summary - WM z-score

Model	R	R²	Adjusted R²	RMSE
M_0	0.000	0.000	0.000	0.752
M_1	0.284	0.081	−0.113	0.794

Note. M_1 includes IG_Frontal, IG_Central, IG_Temporal, IG_Posterior

ANOVA

Model		Sum of Squares	df	Mean Square	F	p
M_1	Regression	1.053	4	0.263	0.418	0.794
	Residual	11.968	19	0.630		
	Total	13.021	23			

Note. M_1 includes IG_Frontal, IG_Central, IG_Temporal, IG_Posterior
Note. The intercept model is omitted, as no meaningful information can be shown.

Coefficients

Model		Unstandardized	Standard Error	Standardized	t	p
M_0	(Intercept)	-2.266×10^{-17}	0.154		-1.476×10^{-16}	1.000
M_1	(Intercept)	−0.443	1.411		−0.314	0.757
	IG_Frontal	−27.026	21.618	−0.700	−1.250	0.226
	IG_Central	2.922	23.158	0.064	0.126	0.901
	IG_Temporal	3.321	25.220	0.064	0.132	0.897
	IG_Posterior	24.505	27.035	0.561	0.906	0.376

Linear Regression IG_IR

Model Summary - IR z-score

Model	R	R²	Adjusted R²	RMSE
M_0	0.000	0.000	0.000	0.651
M_1	0.543	0.295	0.146	0.601

Note. M_1 includes IG_Frontal, IG_Central, IG_Temporal, IG_Posterior

ANOVA

Model		Sum of Squares	df	Mean Square	F	p
M_1	Regression	2.869	4	0.717	1.984	0.138
	Residual	6.871	19	0.362		
	Total	9.740	23			

Note. M_1 includes IG_Frontal, IG_Central, IG_Temporal, IG_Posterior
Note. The intercept model is omitted, as no meaningful information can be shown.

Coefficients

Model		Unstandardized	Standard Error	Standardized	t	p
M_0	(Intercept)	-1.700×10^{-17}	0.133		-1.280×10^{-16}	1.000
M_1	(Intercept)	−1.518	1.069		−1.420	0.172
	IG_Frontal	18.693	16.380	0.560	1.141	0.268
	IG_Central	4.066	17.547	0.102	0.232	0.819
	IG_Temporal	31.011	19.109	0.691	1.623	0.121
	IG_Posterior	−41.289	20.485	−1.093	−2.016	0.058

Linear Regression IG_DR*

Model Summary - DR z-score

Model	R	R²	Adjusted R²	RMSE
M_0	0.000	0.000	0.000	0.688
M_1	0.588	0.346	0.208	0.612

Note. M_1 includes IG_Frontal, IG_Central, IG_Temporal, IG_Posterior

ANOVA

Model		Sum of Squares	df	Mean Square	F	p
M_1	Regression	3.765	4	0.941	2.509	0.076
	Residual	7.127	19	0.375		
	Total	10.892	23			

Note. M_1 includes IG_Frontal, IG_Central, IG_Temporal, IG_Posterior
Note. The intercept model is omitted, as no meaningful information can be shown.

Coefficients

Model		Unstandardized	Standard Error	Standardized	t	p
M_0	(Intercept)	-2.266×10^{-17}	0.140		-1.613×10^{-16}	1.000
M_1	(Intercept)	−2.656	1.089		−2.440	0.025
	IG_Frontal	2.007	16.682	0.057	0.120	0.906
	IG_Central	−10.467	17.871	−0.249	−0.586	0.565
	IG_Temporal	50.102	19.462	1.056	2.574	0.019
	IG_Posterior	−19.597	20.862	−0.490	−0.939	0.359

Linear Regression hG_WM

Model Summary - WM z-score

Model	R	R²	Adjusted R²	RMSE
H_0	0.000	0.000	0.000	0.752
H_1	0.335	0.112	−0.075	0.780

ANOVA

Model		Sum of Squares	df	Mean Square	F	p
H_1	Regression	1.461	4	0.365	0.600	0.667
	Residual	11.560	19	0.608		
	Total	13.021	23			

Note. The intercept model is omitted, as no meaningful information can be shown.

Coefficients

Model		Unstandardized	Standard Error	Standardized	t	p
H_0	(Intercept)	-2.266×10^{-17}	0.154		-1.476×10^{-16}	1.000
H_1	(Intercept)	0.454	0.986		0.460	0.651
	hG_Frontal	−23.942	26.512	−0.392	−0.903	0.378
	hG_Central	−8.202	31.152	−0.141	−0.263	0.795
	hG_Temporal	22.482	26.072	0.401	0.862	0.399
	hG_Posterior	−0.152	29.024	−0.002	−0.005	0.996

Linear Regression hG_IR**

Model Summary - IR z-score

Model	R	R^2	Adjusted R^2	RMSE
M_0	0.000	0.000	0.000	0.651
M_1	0.659	0.434	0.315	0.538

Note. M_1 includes hG_Frontal, hG_Central, hG_Temporal, hG_Posterior

ANOVA

Model		Sum of Squares	df	Mean Square	F	p
M_1	Regression	4.231	4	1.058	3.649	0.023
	Residual	5.509	19	0.290		
	Total	9.740	23			

Note. M_1 includes hG_Frontal, hG_Central, hG_Temporal, hG_Posterior
Note. The intercept model is omitted, as no meaningful information can be shown.

Coefficients

							95% CI		Collinearity Statistics	
Model		Unstandardized	Standard Error	Standardized	t	p	Lower	Upper	Tolerance	VIF
M_0	(Intercept)	-1.700×10^{-17}	0.133		-1.280×10^{-16}	1.000	−0.275	0.275		
M_1	(Intercept)	−0.281	0.681		−0.412	0.685	−1.705	1.144		
	hG_Frontal	49.996	18.302	0.946	2.732	0.013	11.689	88.302	0.248	4.029
	hG_Central	−48.675	21.505	−0.969	−2.263	0.036	−93.686	−3.664	0.163	6.153
	hG_Temporal	47.482	17.998	0.980	2.638	0.016	9.811	85.153	0.216	4.632
	hG_Posterior	−54.574	20.036	−0.894	−2.724	0.013	−96.511	−12.637	0.276	3.620

Bootstrap Coefficients

						95% CI*	
Model		Unstandardized	Bias	Standard Error	p*	Lower	Upper
M_0	(Intercept)	−0.002	−0.001	0.131	0.988	−0.247	0.272
M_1	(Intercept)	−0.310	−0.034	0.674	0.671	−1.559	1.202
	hG_Frontal	49.504	0.506	21.161	0.003	16.263	103.425
	hG_Central	−48.416	−5.576	28.190	0.020	−107.850	−9.045
	hG_Temporal	48.864	3.770	23.946	0.044	2.434	89.897
	hG_Posterior	−53.036	1.653	20.486	0.004	−101.558	−19.262

Note. Bootstrapping based on 5000 replicates.
Note. Coefficient estimate is based on the median of the bootstrap distribution.
* Bias corrected accelerated.

Linear Regression hG_DR**

Model Summary - DR z-score

Model	R	R²	Adjusted R²	RMSE
M_0	0.000	0.000	0.000	0.688
M_1	0.664	0.441	0.324	0.566

Note. M_1 includes hG_Frontal, hG_Central, hG_Temporal, hG_Posterior

ANOVA

Model		Sum of Squares	df	Mean Square	F	p
M_1	Regression	4.808	4	1.202	3.755	0.021
	Residual	6.083	19	0.320		
	Total	10.892	23			

Note. M_1 includes hG_Frontal, hG_Central, hG_Temporal, hG_Posterior
Note. The intercept model is omitted, as no meaningful information can be shown.

Coefficients

							95% CI		Collinearity Statistics	
Model		Unstandardized	Standard Error	Standardized	t	p	Lower	Upper	Tolerance	VIF
M_0	(Intercept)	-2.266×10^{-17}	0.140		-1.613×10^{-16}	1.000	−0.291	0.291		
M_1	(Intercept)	−0.570	0.715		−0.797	0.435	−2.067	0.927		
	hG_Frontal	47.334	19.233	0.847	2.461	0.024	7.079	87.589	0.248	4.029
	hG_Central	−59.893	22.599	−1.127	−2.650	0.016	−107.194	−12.593	0.163	6.153
	hG_Temporal	58.625	18.914	1.144	3.100	0.006	19.038	98.212	0.216	4.632
	hG_Posterior	−47.996	21.055	−0.744	−2.280	0.034	−92.065	−3.926	0.276	3.620

Bootstrap Coefficients

						95% CI*	
Model		Unstandardized	Bias	Standard Error	p*	Lower	Upper
M_0	(Intercept)	−0.001	0.002	0.137	0.992	−0.248	0.289
M_1	(Intercept)	−0.577	−0.041	0.913	0.478	−2.497	1.238
	hG_Frontal	48.422	2.629	23.489	0.025	5.638	95.404
	hG_Central	−59.032	−2.850	22.004	0.008	−117.372	−30.551
	hG_Temporal	59.281	0.799	17.229	0.018	19.089	90.073
	hG_Posterior	−47.415	−0.015	21.094	0.028	−90.182	−7.915

Note. Bootstrapping based on 5000 replicates.
Note. Coefficient estimate is based on the median of the bootstrap distribution.
* Bias corrected accelerated.
