# Supplementary material for: Resting-state EEG gamma power predicts immediate and delayed recall in healthy adults
Source: Cogn Neurodyn. 2025 Aug 26;19(1):138. doi: 10.1007/s11571-025-10313-2 (PMC12381311; doi:10.1007/s11571-025-10313-2)
Supplement: Supplementary file 3 — Supplementary Material 3 [file 11571_2025_10313_MOESM3_ESM.pdf]

Results

Linear Regression TG PAC_WM

Model Summary - WM z-score

Model	R	R²	Adjusted R²	RMSE
H_0	0.000	0.000	0.000	0.752
H_1	0.436	0.191	0.020	0.745

ANOVA

Model		Sum of Squares	df	Mean Square	F	p
H_1	Regression	2.481	4	0.620	1.118	0.377
	Residual	10.543	19	0.555		
	Total	13.024	23			

Note. The intercept model is omitted, as no meaningful information can be shown.

Coefficients

Model		Unstandardized	Standard Error	Standardized	t	p
H_0	(Intercept)	-8.333×10^{-4}	0.154		−0.005	0.996
H_1	(Intercept)	−2.078	1.186		−1.752	0.096
	TG_Frontal	16.912	33.823	0.236	0.500	0.623
	TG_Central	44.677	30.058	0.689	1.486	0.154
	TG_Temporal	−11.702	28.704	−0.174	−0.408	0.688
	TG_Posterior	−23.749	31.369	−0.391	−0.757	0.458

Linear Regression TG PAC_IR

Model Summary - IR z-score

Model	R	R^2	Adjusted R^2	RMSE
H_0	0.000	0.000	0.000	0.651
H_1	0.418	0.175	0.001	0.651

ANOVA

Model		Sum of Squares	df	Mean Square	F	p
H_1	Regression	1.706	4	0.426	1.006	0.429
	Residual	8.052	19	0.424		
	Total	9.758	23			

Note. The intercept model is omitted, as no meaningful information can be shown.

Coefficients

Model		Unstandardized	Standard Error	Standardized	t	p
H_0	(Intercept)	2.833×10^{-17}	0.133		2.131×10^{-16}	1.000
H_1	(Intercept)	−1.075	1.037		−1.037	0.313
	TG_Frontal	25.670	29.559	0.414	0.868	0.396
	TG_Central	41.250	26.268	0.735	1.570	0.133
	TG_Temporal	−10.891	25.086	−0.188	−0.434	0.669
	TG_Posterior	−44.419	27.414	−0.845	−1.620	0.122

Linear Regression TG PAC_DR

Model Summary - DR z-score

Model	R	R²	Adjusted R²	RMSE
H_0	0.000	0.000	0.000	0.688
H_1	0.476	0.227	0.064	0.666

ANOVA

Model		Sum of Squares	df	Mean Square	F	p
H_1	Regression	2.470	4	0.617	1.393	0.274
	Residual	8.422	19	0.443		
	Total	10.892	23			

Note. The intercept model is omitted, as no meaningful information can be shown.

Coefficients

Model		Unstandardized	Standard Error	Standardized	t	p
H_0	(Intercept)	-2.266×10^{-17}	0.140		-1.613×10^{-16}	1.000
H_1	(Intercept)	−1.204	1.060		−1.136	0.270
	TG_Frontal	51.790	30.230	0.791	1.713	0.103
	TG_Central	23.526	26.865	0.397	0.876	0.392
	TG_Temporal	2.743	25.656	0.045	0.107	0.916
	TG_Posterior	−64.289	28.037	−1.158	−2.293	0.033
